# Prediction of Cognitive Decline Using Heart Rate Fragmentation Analysis: The Multi-Ethnic Study of Atherosclerosis

**DOI:** 10.3389/fnagi.2021.708130

**Published:** 2021-08-26

**Authors:** Madalena D. Costa, Susan Redline, Timothy M. Hughes, Susan R. Heckbert, Ary L. Goldberger

**Affiliations:** ^1^Margret and H. A. Rey Institute for Non-linear Dynamics in Medicine, Department of Medicine, Beth Israel Deaconess Medical Center, Harvard Medical School, Boston, MA, United States; ^2^Division of Sleep and Circadian Disorders, Department of Medicine and Neurology, Brigham and Women’s Hospital, Boston, MA, United States; ^3^Department of Medicine, Beth Israel Deaconess Medical Center, Harvard Medical School, Boston, MA, United States; ^4^Section on Gerontology and Geriatric Medicine, Department of Internal Medicine, Wake Forest School of Medicine, Winston-Salem, NC, United States; ^5^Department of Epidemiology and Prevention, Wake Forest School of Medicine, Winston-Salem, NC, United States; ^6^Department of Epidemiology, University of Washington, Seattle, WA, United States

**Keywords:** aging, autonomic nervous system, cognitive status, dementia, heart rate fragmentation and heart rate variability

## Abstract

**Background:** Heart rate fragmentation (HRF), a new non-invasive metric quantifying cardiac neuroautonomic function, is associated with increasing age and cardiovascular disease. Since these are risk factors for cognitive decline and dementia, in the Multi-Ethnic Study of Atherosclerosis (MESA), we investigated whether disrupted cardiac neuroautonomic function, evidenced by increased HRF, would be associated with worse cognitive function assessed concurrently and at a later examination, and with greater cognitive decline.

**Methods:** HRF was derived from the ECG channel of the polysomnographic recordings obtained in an ancillary study (*n* = 1,897) conducted in conjunction with MESA *exam 5* (2010–2012). Cognitive function was assessed at *exam 5* and 6.4 ± 0.5 years later at *exam 6* (2016–2018) with tests of global cognitive performance (the Cognitive Abilities Screening Instrument, CASI), processing speed (Digit Symbol Coding, DSC) and working memory (Digit Span). Multivariable regression models were used to quantify the associations between HRF indices and cognitive scores.

**Results:** The participants’ mean age was 68 ± 9 years (54% female). Higher HRF at baseline was independently associated with lower cognitive scores at both *exams 5* and *6*. Specifically, in cross-sectional analyses, a one-standard deviation (SD) (13.7%) increase in HRF was associated with a 0.51 (95% CI: 0.17–0.86) points reduction in CASI and a 1.12 (0.34–1.90) points reduction in DSC. Quantitatively similar effects were obtained in longitudinal analyses. A one-SD increase in HRF was associated with a 0.44 (0.03–0.86) and a 1.04 (0.28–1.81) points reduction in CASI and DSC from exams 5 to 6, respectively. HRF added predictive value to the Cardiovascular Risk Factors, Aging, and Incidence of Dementia (CAIDE-APOE-ε4) risk score and to models adjusted for serum concentration of NT-proBNP, an analyte associated with cognitive impairment and dementia.

**Conclusion:** Increased HRF assessed during sleep was independently associated with diminished cognitive performance (concurrent and future) and with greater cognitive decline. These findings lend support to the links between cardiac neuroautonomic regulation and cognitive function. As a non-invasive, repeatable and inexpensive probe, HRF technology may be useful in monitoring cognitive status, predicting risk of dementia and assessing therapeutic interventions.

## Introduction

Risk assessment of cognitive impairment is a major public health priority, especially given the increasing prevalence of dementia syndromes in the aging population. A key barrier to progress is the lack of reliable, quantitative and non-invasive methods that complement expensive brain imaging technologies, semi-quantitative functional tests of cognitive status as well as emerging biochemical and “omics” probes. Important insights into the pathophysiology of cognitive impairment syndromes derive from increasing evidence linking central nervous system dysfunction with perturbations in autonomic function (Collins et al., 2012; Fanciulli et al., 2013; Meel-van den Abeelen et al., 2013; Tahsili-Fahadan and Geocadin, 2017; Ahmed et al., 2018). The major probes of such interactions have been heart rate variability (HRV) measures. A PubMed search for “(heart rate variability) AND (cognitive function)” yields over 1,000 citations. Despite this large body of work, traditional HRV measures have failed to gain traction in clinical practice as reliable predictors of mild cognitive impairment or of major adverse cardiovascular events (CVEs), which are strong correlates of cognitive decline and dementia syndromes (Zlokovic et al., 2020).

The failure of traditional HRV indices to fulfill their promise as translational, non-invasive probes of cardiac neuroautonomic function is ascribable to its fundamental assumption, one that equates the *amplitude* of variations in sinus normal-to-normal (NN) intervals with the degree of vagal tone modulation (HRV, 1996). With aging and organic heart disease, physiologic vagal activity almost invariably decreases. In these contexts, a consistent reduction in short-term HRV values would be expected. However, multiple studies (Reardon and Malik, 1996; Bruyne et al., 1999; Stein et al., 2005; Huikuri and Stein, 2012; Drawz et al., 2013; Raman et al., 2017) have reported a counterintuitive increase in the amounts of short-term/high-frequency (0.15–0.4 Hz) variability in high-risk individuals. We (Costa et al., 2017a) have referred to the occurrence of high HRV in settings where highly diminished vagal tone modulation is anticipated as the “HRV paradox.”

Recently, we (Costa et al., 2017a,b) delineated a property of heart rate (HR) dynamics, termed *heart rate fragmentation (HRF)*, which helps resolve this paradox. Heart rate fragmentation metrics quantify variations in NN intervals due to non-respiratory sinus arrhythmia and other anomalous dynamical variants. We previously reported that: (i) HRF monotonically increases with cross-sectional age both in healthy subjects and in those with coronary artery disease (Costa et al., 2017a,b); (ii) increased HRF is associated with both incident major adverse CVEs (Costa et al., 2018) and incident atrial fibrillation (AF) (Costa et al., 2021) in the Multi-Ethnic Study of Atherosclerosis (MESA), and (iii) HRF metrics add significant predictive value to cardiac risk indices such as Framingham (D’Agostino et al., 2008; Costa et al., 2018) and CHARGE-AF^1^ (Alonso et al., 2013; Costa et al., 2021), in MESA. These findings motivated the present investigation. Our working hypotheses were that disrupted cardiac neuroautonomic function at a baseline time, evidenced by increased degree of HRF, would be independently associated with (1) lower cognitive performance, measured concurrently and at a future time, and (2) greater longitudinal cognitive decline in a cohort of participants in MESA. Secondarily, we hypothesized that HRF metrics would be inversely associated with cognitive test scores in models with serum concentration of NH_2_-terminal prohormone B-type natriuretic peptide (NT-proBNP), a major cardiovascular (CV) risk factor also reported to be associated with dementia (Ostovaneh et al., 2020), cognitive performance (Ferguson et al., 2018; Ostovaneh et al., 2020) and brain structure and function by MRI (Zonneveld et al., 2017; Ferguson et al., 2018).

We included widely-used HRV metrics in this study to contrast their performances with those of the HRF indices. Within the canonical HRV interpretative framework, in which increased amount of high-frequency variability is a reflection of increased parasympathetic activity, positive cross-sectional and longitudinal relationships would be expected between the HRV indices and the cognitive scores. However, we did not anticipate finding consistent associations due to the fact that MESA is a study of middle-age to older participants, which increases the probability that the observed variability is due to HRF rather than vagal tone modulation.

## Materials and Methods

### Study Population and Data Collection

The MESA study has been previously described in detail (Bild et al., 2002). Briefly, in 2000–2002, 6,814 persons between the ages of 45 and 84 without clinically evident CV disease were recruited at six U.S. field centers. Institutional review boards approved the conduct of the study. Written informed consent was obtained from all participants. MESA fifth examination, conducted in 2010–2012, included 4,656 participants. MESA sixth examination, conducted in 2016–2018, included 3,303 participants, 95% (3,153) of whom had also participated in *exam 5*.

The HR dynamical indices used here (HRF and HRV) were obtained from an ancillary sleep study conducted in 2010–2013 in conjunction with MESA’s fifth examination. The study included 2,237 (out of 4,656) participants, who underwent in-home overnight polysomnography (PSG) following a standardized protocol (Chen et al., 2015). Two thousand and fifty seven participants had a valid PSG study. Hereafter, we refer to this group as the MESA-Sleep cohort. Compared with the participants who did not undergo the sleep examination, those who did and had a valid study were relatively younger (mean, 68 vs. 71 yr), less likely to be hypertensive (58 vs. 62%), and more likely to be Hispanic (23.5% vs. 19.1%). They had lower coronary artery calcification (CAC) scores (median [interquartile range]: 30 [0–215] vs. 57 [0–338]) and lower blood concentration of NT-proBNP (71 [35–139] vs. 83 [43–181] pg/ml). There were no differences in sex, body mass index, smoking status, diabetes and prevalent CV disease. Similarly, there were no differences in the prevalence of self-report doctor-diagnosed sleep apnea.

The PSG data were scored at a centralized sleep reading center in accordance with published guidelines (Redline et al., 1998, 2007). The electrocardiographic (ECG) channel, sampled at 256 Hz, was processed using Compumedics Somte software (Compumedics LTd., Abbottsville, Australia) for detection and classification of the QRS complexes as normal sinus (N), premature ventricular complexes or premature supraventricular complexes. The automated annotations were reviewed and corrected when necessary by a trained technician. One hundred and sixty participants were excluded due to: poor signal quality (*n* = 33), electronic pacemaker (*n* = 14), missing annotations for sleep stage or QRS complexes (*n* = 14), <2 h of combined sleep periods scored as rapid eye movement (REM) or non-REM (*n* = 14), in AF at the time of the PSG (*n* = 22), prevalent dementia as defined below (*n* = 23), missing information on dementia status (*n* = 29) and those for whom (*n* = 11) the last recorded follow-up for AF events was prior to the PSG study. Overall, HR dynamical measures were calculated for 1,897 participants.

### Clinical Follow-Up and Event Classification

In addition to clinical exams, MESA participants were contacted by telephone every nine to twelve months to obtain information about hospital admissions and medical events. For those over age 65 and enrolled in fee-for-service Medicare, claims data were also used to identify diagnosis and procedure codes. Trained personnel abstracted any hospital records suggesting possible CVEs, which were then adjudicated by the MESA Morbidity and Mortality Committee. Non-fatal endpoints in MESA include congestive heart failure, angina, myocardial infarction, percutaneous coronary intervention, coronary bypass grafting or other revascularization procedure, resuscitated cardiac arrest, peripheral arterial disease, stroke (non-hemorrhagic) and transient ischemic attack. Cardiovascular deaths, as adjudicated by committee review, included fatalities directly related to stroke or coronary heart disease. The definition and adjudication of these events have been previously described (Bild et al., 2002; Bluemke et al., 2008; Yeboah et al., 2012). The identification of prevalent AF (Heckbert et al., 2017), including fibrillation and flutter, was based on diagnostic codes from hospital admissions, inpatient and outpatient Medicare claims (for those enrolled in fee-for-service Medicare) and study 12-lead ECGs. Dementia diagnosis was based on death certificates and hospital discharge ICD codes (Fujiyoshi et al., 2017) in addition to usage of acetylcholinesterase inhibitor and other medications used specifically for the treatment of Alzheimer’s disease and related dementias.

### Assessment of Cognitive Performance

In MESA (Fitzpatrick et al., 2015), three different standardized and validated tests were used to quantify cognitive performance: the Cognitive Abilities Screening Instrument (CASI, version 2) (Teng et al., 1994), the digit symbol coding (DSC) (Wechsler, 1997), and the digit span (DS) (Wechsler, 1997). The CASI is a test of global cognitive function that contains 25 items representing nine cognitive domains, including short- and long-term memory, attention, concentration, orientation, language, verbal fluency, visual construction, and abstraction/judgment. Scores from individual items on the CASI were summed to obtain an overall cognitive function score (range 0–100). The DSC (range 0–133) is a subtest of the Wechsler Adult Intelligence Scale III (WAIS-III). Participants are given a random sequence of digits (1–9) and asked to write the correct symbol (e.g., +, >) for each digit based on a legend of digit–symbol pairs. The score is the number of correct symbols written in 120 seconds. The DSC score quantifies processing speed (how quickly simple perceptual or mental operations can be performed). The DS, also a subtest of WAIS-III, consists of two tasks. In one, participants are asked to repeat forward and in the other backward, increasingly longer spans of randomly ordered digits read to them. The scores are based on the total number of correct answers (DS forward: range 0–16; DS backward: range, 0–14). The DS forward and backward measure related but slightly different aspects of memory (Ramsay and Reynolds, 1995; Cullum, 1998; Izawa et al., 2009). The DS forward is assumed to assess mainly attention and short-term auditory memory. The DS backward is assumed to measure working memory subsuming short-term memory and elements of executive control required to manipulate verbal information. Higher CASI, DSC and DS scores are indicative of better cognitive performance.

We excluded participants with missing or invalid cognitive scores due to physical (hearing, vision, motor) impairments that interfere with performance, language barriers, and circumstantial reasons, such as answering a cell phone call during a test. In the case of CASI, tests with a score <20 (*n* = 12) and those missing responses to more than three questions (*n* = 9) were also considered invalid. Overall, analyses with *exam 5* CASI, DS forward, DS backward and DSC scores included 1,823 (96%), 1,881 (99%), 1,884 (99%) and 1,715 (90%) of the 1,897 participants for whom HR dynamical indices were calculated. Participants in the MESA-Sleep cohort with missing or invalid *vs.* valid DSC scores were on average two years older, had significantly higher systolic blood pressure (BP) and body mass index (BMI) values. A higher percentage had had a CVE (12.5 *vs*. 8.6%). Their degree of HRF was significantly higher (PNNSS: 68.3 vs. 65.6%).

Approximately 62% (2,051 out of 3,303) of participants in MESA *exam 6* underwent cognitive evaluation. The number of participants with a PSG study and valid cognitive scores at *exams 5* and *6* was 971, 1016 and 845 for the CASI, DS and DSC tests, respectively.

### Assessment of Baseline Covariates at MESA Fifth Examination (2010–2012)

Age, sex, race/ethnicity (White, Chinese-American, African-American and Hispanic) smoking status, and medication use were self-reported. Systolic and diastolic BPs were measured three times after the participant rested for five minutes; the means of the last two measurements were used here. Diabetes was defined by a fasting blood glucose ≥126 mg/dL or self-reported use of antidiabetic medication. Antihypertensive medication included beta-blockers, calcium channel blockers, angiotensin converting enzyme inhibitors or receptor blockers, vasodilators and diuretics. Depression was defined as a score of more than 15 points in the Center of Epidemiological Studies Depression scale. Antidepressant medication included tricyclic and non-tricyclic (other than monoamine oxidase inhibitors) medications accompanied or not by antipsychotic medications. Alcohol use (a binary variable: yes/no) was defined as consuming ≥ 1 glass of wine or the equivalent per week. Physical activity, defined as the number of metabolic equivalent minutes per week of intentional moderate and vigorous activity, was quantified using the MESA Typical Week Physical Activity Survey, adapted from the Cross-Cultural Activity Participation Study (Bertoni et al., 2009). APOE isoforms were estimated from single nucleotide polymorphisms rs429358 and rs7412 (Fitzpatrick et al., 2015). Gross family income was dichotomized as below and above $75,000 (top quartile). Sleep maintenance efficiency was defined as the percentage of sleep time asleep after sleep onset. Two measures of sleep structure included in this study were the percentage of time in sleep stages N3 (slow wave sleep [SWS]) and the percentage of time in REM. Sleep disordered breathing was assessed by the apnea hypopnea index (AHI), defined as the average number per hour of sleep of all apneas and hypopneas associated with ≥3% oxygen desaturation or arousal, and two measures of overnight hypoxemia: the percentage of sleep time with pulse oximeter oxygen saturation <90% (% time SpO_2_ < 90%), and the oxygen desaturation index (ODI), defined as the number of oxygen desaturations ≥ 3% per hour of sleep. The CAC scores, derived from computed tomography imaging using the Agatston method (Agatston et al., 1990), was available for 1,464 (out of 1,897; 77%) participants at *exam 5* (Carr et al., 2005). There were no significant differences in demographic (age, sex, race) and CV risk factors (diabetes, pack-years of smoking, hypertensive status, BMI, CV and AF disease prevalence) between those with and without CAC scores. Serum concentration of NT-proBNP measured at *exam 5* was available for all but 39 participants in our study.

### Data Analyses

The ECG channels of the PSG recordings obtained at MESA *exam 5* were analyzed for detection and classification of each QRS complex as normal (N) sinus, premature supraventricular or premature ventricular complexes. The sequence of normal-to-normal (NN) intervals between sleep onset and sleep termination were extracted for computation of HRF and traditional HRV indices.

#### Heart Rate Fragmentation Metrics

Heart rate fragmentation was quantified by an ensemble of statistical metrics derived from the analysis of NN interval time series, as described in Costa et al. (2017a, 2018). These metrics are based on counts of HR acceleration, deceleration and no-change intervals. Let *t*_*i*_ represent the time of occurrence of a given QRS complex and NN_*i*_ the time interval (*t_*i*_ – t_*i–*__1_*) between consecutive QRS complexes. Heart rate acceleration, deceleration and no-change intervals (in seconds) are defined as ΔNN_*i*_ ≤ –*n/*SF, ΔNN_*i*_ ≥ *n/*SF and –*n/*SF < ΔNN_*i*_ < *n/*SF, respectively, where ΔNN_*i*_ = NN*_*i*_ –* NN*_*i–*_*_1_, *n* is a positive integer (in this case, *n* = 1) and SF is the sampling frequency (in Hz) of the ECG signal. (Note that HR and ΔNN_*i*_ values are inversely related.) Sequences of negative (positive) ΔNN intervals are termed accelerative (decelerative) segments. The length of a segment is the number of ΔNN intervals it contains.

We computed three inter-related indices of overall degree of fragmentation (PIP, PNNLS, PNNSS). Briefly, PIP, the percentage of “inflection points,” is defined as the combined percentage of transitions from HR acceleration to HR deceleration and vice-versa (“hard” inflection points), and from HR acceleration/deceleration to no-change in HR and vice-versa (“soft” inflection points). Mathematically, a given NN_*i*_ interval is an inflection point if ΔNN_*i*__+__1_ ×ΔNN_*i*_ ≤ 0 and ΔNN_*i*__+__1_ ≠ΔNN_*i*_. The overall percentage of ΔNN intervals in long segments, PNNLS, is the number of ΔNN intervals in accelerative/decelerative segments with ≥3 ΔNN intervals over the total number of ΔNN intervals. The percentage of ΔNN intervals in short segments, PNNSS, is the number of ΔNN intervals in accelerative/decelerative segments with <3 ΔNN intervals over the number of ΔNN intervals in accelerative/decelerative segments of any length. More fragmented time series have higher PIP and PNNSS values, and lower PNNLS values.

#### Traditional HRV Indices

Traditional time domain HRV indices (HRV, 1996) were calculated from NN interval time series between sleep onset and sleep termination using a 5-min sliding window (without overlap). Windows with <150 beats and/or >75% NN intervals were excluded. The following time domain measures were computed: (1) the average of all NN intervals (AVNN), (2) the mean of the standard deviations (SDs) of NN intervals in all qualified, 5-min window (SDNN), and (3) the root mean square of successive NN interval differences (rMSSD). On the frequency domain, we calculated the metric high-frequency (HF) power defined as the total spectral power of NN intervals between 0.15 and 0.4 Hz. Power spectrum estimates were obtained using the Lomb periodogram method. For each subject, the values from the different windows were averaged. Of note, mean HR is not a measure of variability of the fluctuations in HR. However, AVNN, which is inversely related to mean HR, is generally included in the set of indices that became known as traditional HRV measures. Here, we use the term HRV to refer to the *method* of time series analysis and HR variability to refer to the *amplitude of the fluctuations* (variability) in HR. (Mean HR is an HRV measure but not a measure of HR variability.) The source codes for HRV computations are available at www.physionet.org (Goldberger et al., 2000).

### Specific Hypotheses

Participants with higher HRF, i.e., higher PIP and PNNSS values and lower PNNLS values, at baseline (*exam 5*) were hypothesized to have (1) lower cognitive scores both at baseline and at *exam 6*, and (2) larger decreases in cognitive scores from *exams 5* to *6*.

### Statistical Analyses

This presentation includes both cross-sectional and longitudinal analyses. The former used MESA-Sleep (*exam 5*) data. The latter used MESA-Sleep data, our baseline, and MESA *exam 6* data obtained 6.4 ± 0.5 (mean ± SD) years later. (In the longitudinal studies only participants with both cognitive testing at exams 5 and 6 were included.) Continuous variables are summarized as median and interquartile range, unless otherwise indicated. Categorical variables are presented as numbers and percentages. Differences in baseline characteristics between those with and without cognitive testing at *exam 6* were evaluated using the χ^2^ and Mann-Whitney tests for categorical and continuous variables, respectively. Differences in baseline characteristics across quartiles of PNNSS were assessed using the χ^2^ and the ANOVA tests for categorical and continuous variables, respectively. Variables with skewed distributions (rMSSD, SDNN, HF, weekly amount of moderate and vigorous physical activity, serum concentration of NT-proBNP, pack-years of cigarette smoking, % time SpO_2_ < 90%, ODI, and CAC score) were transformed using the natural logarithm. In the case of the last three measures, we added 1 to the variables’ values before the logarithmic transformation due to the occurrence of 0 values.

We used multivariable linear regression models to quantify the associations between cognitive test scores and HR dynamical indices. In the text of the manuscript, we show the results for five models. *Model 1* was unadjusted. *Model 2* was adjusted for the Cardiovascular Risk Factors, Aging, and Incidence of Dementia (CAIDE) Risk Score with APOE-ε4 (CAIDE–APOE-ε4), a validated tool to predict late-life dementia risk (20 years later) (Kivipelto et al., 2006; Exalto et al., 2014). *Model 3* was adjusted for age, race/ethnicity, sex, and education level. *Model 4*, hereafter referred to as “fully adjusted,” included the variables of *Model 3* in addition to APOE-ε4, CV risk factors (usage of anti-hypertensive and lipid lowering medications, systolic BP, diabetes mellitus status, total cholesterol, high-density lipoprotein cholesterol, pack-years of cigarette smoking), alcohol consumption, weekly amounts of moderate and vigorous physical activity, depression, usage of antidepressant medications, total gross family income ≥ $75,000, mean HR and prevalent CV and AF events. *Model 5*, was further adjusted for NT-proBNP (Zonneveld et al., 2017; Ferguson et al., 2018; Nagata et al., 2019; Ostovaneh et al., 2020). Finally, we considered models that included the variables in *Model 4* and one of the following variables previously reported to be associated with overall or specific domains of cognitive function: CAC scores (Bos et al., 2015; Deckers et al., 2017), % SWS (Wunderlin et al., 2020) and % REM (Djonlagic et al., 2021), % time SpO_2_ < 90% (Yaffe et al., 2011; Johnson et al., 2017) and ODI (Johnson et al., 2017).

Continuous independent variables were standardized. Thus, the regression coefficients presented in Tables 3–6 indicate the expected difference in cognitive scores per one-SD increment in the independent variable. Multivariable linear regression models were also used to quantify the associations between the baseline HRF indices and the changes in cognitive test scores from baseline to *exam 6* (Δ = *exam 6* – *exam 5*). These analyses were adjusted for the covariates detailed above in addition to baseline cognitive test scores. Of note, the regression of Δ*Y* = *Y*_2_– *Y*_1_ on both *Y*_1_ and *X* is equivalent to the regression of *Y*_2_ on *Y*_1_ and *X* (Werts and Linn, 1970). Standardized regression coefficients (where only the independent variables were standardized) are presented.

The likelihood ratio test was used to evaluate the difference in performance between two nested models. The larger model contained an HRF index in addition to the variables in the base model. A statistically significant result for the likelihood ratio test indicates that the addition of the HRF index to the base model significantly increased its fit, i.e., its predictive value. Statistical significance was set at a *p*-value <0.05. All *p*-values reported were two-sided. A *p*-value <0.1 was considered borderline significant. All analyses were performed using STATA software (version12.0 for Linux).

## Results

### Baseline Characteristics of the MESA Participants

Participants in the MESA-Sleep cohort compared to those enrolled in *exam 5* who did not undergo the sleep examination were relatively younger (mean, 68 *vs.* 71 years), less likely to be hypertensive (58% *vs.* 62%), and more likely to be Hispanic (23.5% *vs.* 19.1%) [19]. They had lower CAC scores (median [inter-quartile range]: 30 [0–215] *vs.* 57 [0–338]) and higher DSC scores (52 [39–65] *vs.* 50 [37–62]). There were no differences in sex, BMI, smoking status, diabetes, number of participants with prevalent CV disease, and in the results of the two other cognitive tests, CASI and the DS.

Approximately 50% of the participants in the MESA-Sleep cohort underwent cognitive testing at *exam 6*. Table 1 summarizes demographic, clinical and HR dynamical characteristics of participants in the MESA-Sleep cohort, and in the subgroups of those with (*subgroup A*) and without (*subgroup B*) cognitive testing at *exam 6*. Participants in *subgroup* A had higher cognitive test scores at *exam 5*. They were approximately three years younger and healthier overall.

**TABLE 1:** Values at *exam 5* of demographic, clinical and other characteristics of participants in the study cohort and in the subgroups of those with and without cognitive tests at *exam 6*.

Values at Exam 5						

Variables			Subgroup A		Subgroup B	
	MESA-Sleep Cohort		With CASI at exam 6		Without CASI at exam 6	
	*N* = 1,897		*N* = 998		*N* = 899	
Age (year)	67	[60–75]	66	[60–73]	69	[61–77]
Male	874	(46.1)	455	(45.6)	419	(46.6)
Race/ethnicity:						
White	685	(36.1)	395	(39.6)	290	(32.3)
Chinese-American	235	(12.4)	120	(12.0)	115	(12.8)
African-American	526	(27.7)	305	(30.6)	221	(24.6)
Hispanic	451	(23.8)	178	(17.8)	273	(30.4)
Education:						
High school or less	571	(30.2)	238	(23.9)	333	(37.0)
Some college	458	(24.2)	252	(25.4)	206	(22.9)
Bachelor degree or higher	864	(45.6)	504	(50.7)	360	(40.0)
Gross household income ≥ 50K	866	(46.9)	529	(54.0)	337	(38.9)
Gross household income ≥ 75K	542	(29.4)	337	(34.4)	205	(23.7)
Systolic blood pressure (mmHg)	120	[109–135]	119	[108–133]	121	[110–136]
Anti-hypertensive medication	983	(51.8)	486	(48.7)	497	(55.3)
Beta blockers	307	(16.2)	142	(14.2)	165	(18.4)
Body mass index (Kg/m^2^)	27.8	[24.7–31.8]	27.8	[24.7–31.7]	27.9	[24.6–31.9]
Total cholesterol (mg/dl)	183	[159–208]	184	[159–210]	183	[158–206]
HDL (mg/dl)	53	[44–64]	53	[45–64]	53	[43–63]
Lipid lowering medication	689	(36.3)	341	(34.2)	348	(38.7)
Physical activity, ln (MET-min/wk)	3690	[1770–7185]	4011	[2033–7710]	3368	[1470–6495]
Depression, CESD ≥ 16	262	(14.2)	123	(12.5)	142	(16.2)
Anti-depression medication	260	(13.9)	142	(14.4)	118	(13.4)
Smoking: current	124	(6.57)	59	(5.94)	61	(7.31)
Pack-years of cigarette smoking, ln	0	[0–2.59]	0	[0–2.51]	0	[0–2.64]
Presently drinking alcohol	822	(43.5)	478	(48.1)	344	(38.5)
Diabetes mellitus	355	(18.9)	162	(16.4)	193	(21.8)
Prevalent CVEs	170	(9.0)	77	(7.7)	93	(10.3)
History of AF	74	(3.9)	34	(3.4)	40	(4.5)
NT-proBNP (pg/ml)	67.53	[34.1–133]	60.07	[30.4–119]	79.82	[38.9–159]
Sleep duration (min)	370	[314–416]	370	[317–416]	370	[310–416]
Sleep maintenance efficiency (%)	82.7	[74.2–89.3]	83	[75.5–89.9]	81	[72.9–88.7]
% REM	18.4	[14.0–22.5]	18	[14.1–22.8]	18	[13.7–22.2]
% SWS	8.4	[2.3–15.8]	8	[2.4–15.8]	8	[2.0–15.8]
% time SpO2 < 90%	0.59	[0.04–3.09]	0.47	[0.03–2.05]	0.75	[0.05–3.76]
AHI, ln (%)	2.94	[2.33–3.52]	2.89	[2.28–3.44]	3.05	[2.42–3.58]
ODI, ln (%)	2.81	[2.11–3.42]	2.73	[2.06–3.37]	2.93	[2.13–3.48]
CASI	89	[83–94]	91	[85–95]	87	[81–93]
Digit symbol coding	52	[40–65]	56	[45–68]	48	[34–61]
Digit span forward	9	[8–12]	10	[8–12]	9	[7–11]
Digit span backward	5	[4–7]	6	[4–7]	5	[4–7]
PIP (%)	58.2	[53.6–63.3]	57.0	[52.7–62.4]	59.3	[54.7–64.4]
PNNSS (%)	66.3	[56.2–76.0]	64.5	[54.3–74.8]	68.0	[58.5–77.5]
PNNLS (%)	30.6	[21.7–39.7]	32.3	[23.2–41.4]	29.0	[20.4–37.8]
AVNN (ms)	942	[861–1033]	943	[862–1031]	940	[860–1034]
rMSSD, ln (ms)	28.6	[20.5–42.0]	28.6	[20.9–41.8]	28.6	[19.9–42.2]
SDNN, ln (ms)	46.3	[35.0–61.2]	46.4	[35.5–62.0]	46.3	[34.3–60.8]
HF, ln (ms^2^)	372	[193–737]	378	[197–726]	365	[189–764]

*Values shown are median [1st – 3rd quartiles] for continuous variables and number of participants (%) for categorical variables.*

*AF, atrial fibrillation; AHI, apnea-hypopnea index; AVNN, average value of NN intervals; CASI, the Cognitive Abilities Screening Instrument score; CESD, Center for Epidemiological Studies Depression scale; CVE, cardiovascular event; HDL, High-density lipoprotein; HF, high-frequency, the total spectral power of all NN intervals between 0.15 and 0.40 Hz; MESA, Multi-Ethnic Study of Atherosclerosis; MET, metabolic equivalent of task; ODI, oxygen desaturation index; PIP, percentage of inflection points; PNNLS, percentage of NN intervals in long (≥3) accelerative/decelerative segments; PNNSS, percentage of NN intervals in short (<3) accelerative/decelerative segments; REM, rapid eye movement; NT-proBNP, N-terminal prohormone B-type natriuretic peptide; rMSSD, root mean square of successive NN interval differences; SDNN, standard deviation of the NN intervals; SpO_2_, pulse oximeter oxygen saturation; SWS, slow wave sleep.*

Table 2 summarizes demographic, clinical and HR dynamical characteristics of the MESA-Sleep cohort per quartiles of the HRF index, PNNSS. Those in the bottom quartiles (less fragmented) were younger, more active and, overall, healthier. Accordingly, they were less likely to be diabetic, to have had a CVE prior to the PSG study or history of AF. A smaller percentage used anti-hypertensive, beta-blocker and/or lipid lowering medications. A higher percentage had attained higher levels of education and belonged to households with higher gross income. Additionally, those with lower HRF had lower NT-proBNP serum concentration, longer sleep duration, higher sleep maintenance efficiency and lower % time SpO2 < 90%. However, they did not differ in terms of sleep structure, AHI and ODI. Participants with lower HRF, had higher CASI, DSC and DS backward scores both at *exams 5* and *6*.

**TABLE 2:** Demographic and clinical characteristics of MESA-Sleep study cohort per quartile of the HRF index PNNSS.

QUARTILES OF HRF (PNNSS)	Q_1_		Q_2_		Q_3_		Q_4_		*p* value
	<56.21		[56.21, 66.25)		[66.30, 76.04)		≥76.08		
Variables	*N* = 475		*N* = 474		*N* = 474		*N* = 474		
Age (year)	65	8	67	9	69	9	71	9	**<0.001**
Male	234	49.26	226	47.68	214	45.15	200	42.19	0.140
Education:									**<0.001**
High school or less	114	24.1	134	28.33	153	32.42	170	35.86	
Some college	116	24.47	115	24.31	101	21.4	126	26.58	
Bachelor degree or higher	244	51.48	224	47.36	218	46.19	178	37.55	
Gross household income ≥ 50K	270	57.94	240	51.72	185	40.22	171	37.5	**<0.001**
Gross household income ≥ 75K	160	34.33	152	32.76	124	27.0	106	23.25	**0.001**
Systolic blood pressure (mmHg)	118.7	17.8	121.1	18.65	124.8	22.42	125.7	20.34	**<0.001**
Anti-hypertensive medication	187	39.37	239	50.42	243	51.27	314	66.24	**<0.001**
Beta blockers	40	8.42	64	13.5	77	16.24	126	26.6	**<0.001**
Body mass index (Kg/m^2^)	28.6	5.3	28.6	5.47	28.5	5.86	28.9	5.61	0.684
Total cholesterol (mg/dl)	188.4	38.1	188.4	37.66	184.4	32.82	177.8	36.69	**<0.001**
HDL (mg/dl)	55.11	16.74	55.63	16.7	55.88	15.14	55.48	16.7	0.910
Lipid lowering medication	142	29.89	174	36.71	168	35.44	205	43.25	**<0.001**
Physical activity, ln (MET-min/wk)	8.272	0.937	8.162	1.051	8.157	1.043	8.014	1.222	**0.004**
Depression, CESD ≥ 16	62	13.36	63	13.4	69	14.87	71	15.24	0.779
Anti-depression medication	56	12.0	59	12.6	73	15.7	72	15.4	0.236
Smoking: current	25	5.30	32	6.78	30	6.37	37	7.86	0.461
Pack-years of cigarette smoking	1.071	1.448	1.17	1.473	1.166	1.466	1.25	1.56	0.332
Presently drinking alcohol	231	48.94	214	45.34	195	41.4	182	38.48	**0.007**
Diabetes	72	15.2	84	18.0	90	19.27	109	23.24	**0.016**
Prevalent CVEs	25	5.26	36	7.59	43	9.07	66	13.92	**<0.001**
Prevalent AF	6	1.26	17	3.60	12	2.55	39	8.32	**<0.001**
NT-proBNP, ln (pg/ml)	3.959	0.96	4.103	0.96	4.34	0.96	4.591	1.11	**0.002**
Sleep duration (min)	365.6	83.0	366.8	74.5	364.7	78.7	352.4	77.35	**0.015**
Sleep maintenance efficiency (%)	81.74	11.79	80.54	12.0	80.0	11.74	78.26	13.0	**<0.001**
% REM	18.5	6.45	18.3	6.63	18.2	6.41	17.5	6.65	0.089
% SWS	10.5	8.91	10.2	9.09	10.4	8.92	9.86	9.27	0.759
% time SpO2 < 90%, ln	0.811	0.89	0.809	0.966	0.849	0.96	0.977	1.141	**0.029**
AHI, ln (%)	3.009	0.85	2.828	0.879	2.852	0.863	2.824	0.869	0.878
ODI, ln (%)	2.83	0.93	2.67	0.963	2.681	0.944	2.678	0.95	0.930
CASI (*exam 5*)	89.42	7.581	89.0	7.90	88.23	7.878	86.25	8.578	**<0.001**
Digit symbol coding (*exam 5*)	56.26	17.14	53.84	18.65	52.09	18.62	46.79	19.12	**<0.001**
Digit span forward (*exam 5*)	9.865	2.761	9.609	2.838	9.669	2.841	9.555	2.833	0.354
Digit span backward (*exam 5*)	5.951	2.50	5.706	2.424	5.618	2.368	5.30	2.368	**<0.001**
CASI (*exam 6*)	91.28	7.18	90.75	6.372	90.05	7.569	87.11	9.576	**<0.001**
Digit symbol coding (*exam 6*)	55.56	17.3	53.0	18.21	49.4	17.75	45.52	17.64	**<0.001**
Digit span forward (*exam 6*)	9.863	2.65	9.524	2.734	9.50	2.70	9.22	2.556	0.058
Digit span backward (*exam 6*)	5.993	2.37	5.60	2.363	5.564	2.188	5.179	2.057	**0.001**
Δ CASI	0.863	5.92	0.325	5.516	−0.07	6.493	−0.73	8.274	0.057
Δ Digit symbol coding	−3.85	11.7	−3.56	10.78	−6.59	13.0	−6.68	10.74	**0.004**
Δ Digit span forward	−0.26	2.10	−0.19	2.257	−0.55	2.279	−0.69	2.40	**0.043**
Δ Digit span backward	−0.24	2.03	−0.19	2.174	−0.34	1.776	−0.40	2.10	0.661

*Values shown are mean and standard deviation for continuous variables and number and percentage of participants for categorical variables. The p values in bold are < 0.05.*

*AF, atrial fibrillation; AHI, apnea-hypopnea index; CASI, the Cognitive Abilities Screening Instrument score; CESD, Center for Epidemiological Studies Depression scale; CVE, cardiovascular event; HDL, high-density lipoprotein; HRF, heart rate fragmentation; MET, metabolic equivalent of task; ODI, oxygen desaturation index; NT-proBNP, N-terminal prohormone B-type natriuretic peptide; PNNSS, percentage of NN intervals in short (<3) accelerative/decelerative segments; REM, rapid eye movement; SpO_2_, pulse oximeter oxygen saturation; SWS, slow wave sleep.*

### Graphs of the Associations of HRF and HRV Indices With Cross-Sectional Age

**FIGURE 1:**
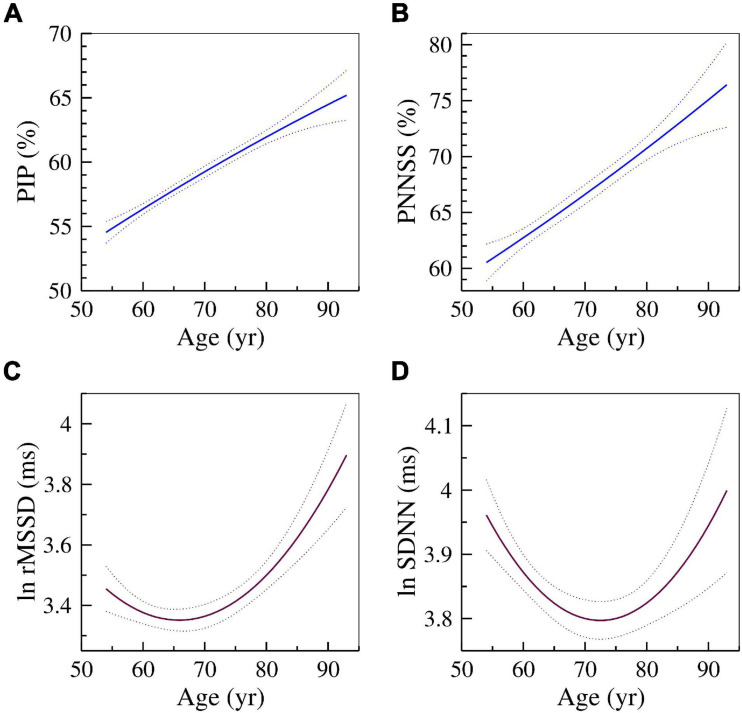
Changes in HRF and HRV with cross-sectional age. The solid and dotted lines are the quadratic fit and 95% confidence intervals obtained from the regressions of the dependent variables **(A)** PIP, **(B)** PNNSS, **(C)** ln rMSSD and **(D)** ln SDNN on age and age^2^.

Figure 1 shows the relationships of two HRF indices, PIP (A) and PNNSS (B), and of two HRV indices, rMSSD (C) and SDNN (D) with cross-sectional age. As expected, HRF indices monotonically increased with cross-sectional age. The measure whose values increase as fragmentation decreases, PNNLS, monotonically decreased with age (not shown). In contrast, the measures of HR variability, rMSSD and SDNN, exhibited a parabolic relationship with cross-sectional age. Variability was higher both for younger and older participants. A similar relationship was observed for HF power (not shown).

### Association of HRF and HRV Indices With Baseline Cognitive Scores

#### Heart Rate Fragmentation

In all models, the three HRF indices were strongly associated with the CASI and the DSC scores (Table 3). The associations were negative for PIP and PNNSS, whose values are higher for more fragmented time series, and positive for PNNLS, whose values are higher for less fragmented (more fluent) time series. Specifically, a one-SD increase in PNNSS (13.7%) was associated with −0.51 (95% CI: −0.86 to −0.17) and −1.12 (−1.90 to −0.34) points decrease in the CASI and DSC scores, respectively, in analyses of *Model 4*. Heart rate fragmentation indices were more strongly associated with the DS backward than the DS forward scores. The associations of HRF indices with DS backward scores were significant in *Models 1–3* and borderline significant in the two most adjusted models. The associations between HRF indices and DS forward scores were only significant in unadjusted analyses and those adjusted for the CAIDE–APOE-ε4 risk index. Further adjusting the previous analyses for NT-proBNP did not qualitatively change any of our results.

**TABLE 3:** Multivariable linear regression analyses of the associations between the HRF indices (PIP, PNNSS and PNNLS) and the cognitive scores (CASI, DSC and DS) measured at *exam 5*.

EXAM 5	MODEL 1				MODEL 2				MODEL 3				MODEL 4				MODEL 5			
	Unadjusted				CAIDE–APOE-ε4				Age, sex, race, education				Demographic + APOE-ε4 + CV				Model 4 + NT-proBNP			
													and other “traditional” risk factors							
	β	95% CI		*p*	β	95% CI		*p*	β	95% CI		*P*	β	95% CI		*p*	β	95% CI		*p*
**CASI**																				
PIP (7.21%)	−1.24	−1.61	−0.88	**<0.001**	−1.12	−1.48	−0.77	**<0.001**	−0.51	−0.83	−0.18	**0.002**	−0.45	−0.80	−0.10	**0.012**	−0.42	−0.77	−0.07	**0.018**
PNNSS (13.7%)	−1.25	−1.63	−0.88	**<0.001**	−1.10	−1.47	−0.74	**<0.001**	−0.47	−0.79	−0.14	**0.005**	−0.51	−0.86	−0.17	**0.004**	−0.48	−0.83	−0.14	**0.006**
PNNLS (12.6%)	1.25	0.88	1.63	**<0.001**	1.11	0.74	1.47	**<0.001**	0.47	0.15	0.79	**0.004**	0.48	0.14	0.82	**0.006**	0.45	0.11	0.80	**0.010**

**DSC**																				

PIP (7.27%)	−3.94	−4.79	−3.08	**<0.001**	−3.57	−4.37	−2.76	**<0.001**	−1.21	−1.98	−0.44	**0.002**	−0.84	−1.64	−0.04	**0.041**	−0.87	−1.68	−0.07	**0.034**
PNNSS (13.8%)	−3.63	−4.49	−2.77	**<0.001**	−3.13	−3.96	−2.30	**<0.001**	−1.17	−1.89	−0.44	**0.002**	−1.12	−1.90	−0.34	**0.005**	−1.17	−1.96	−0.38	**0.004**
PNNLS (12.7%)	3.72	2.86	4.58	**<0.001**	3.23	2.40	4.06	**<0.001**	1.22	0.49	1.95	**0.001**	1.07	0.30	1.84	**0.007**	1.12	0.34	1.90	**0.005**

**DS forward**																				

PIP (7.21%)	−0.16	−0.29	−0.03	**0.014**	−0.12	−0.25	0.01	***0.060***	−0.03	−0.15	0.10	0.682	−0.03	−0.16	0.10	0.663	−0.02	−0.16	0.11	0.738
PNNSS (13.7%)	−0.16	−0.28	−0.03	**0.017**	−0.10	−0.23	0.02	0.110	−0.02	−0.14	0.10	0.732	−0.03	−0.16	0.10	0.630	−0.03	−0.17	0.10	0.629
PNNLS (12.7%)	0.16	0.03	0.28	**0.017**	0.10	−0.02	0.23	0.107	0.02	−0.10	0.13	0.774	0.02	−0.10	0.15	0.713	0.03	−0.11	0.16	0.709

**DS backward**																				

PIP (7.27%)	−0.25	−0.36	−0.15	**<0.001**	−0.22	−0.32	−0.11	**<0.001**	−0.13	−0.23	−0.02	**0.016**	−0.10	−0.21	0.01	***0.068***	−0.09	−0.20	0.02	0.102
PNNSS (13.8%)	−0.26	−0.36	−0.15	**<0.001**	−0.21	−0.32	−0.10	**<0.001**	−0.10	−0.20	0.00	**0.047**	−0.11	−0.22	0.01	***0.065***	−0.10	−0.21	0.01	***0.082***
PNNLS (12.7%)	0.25	0.15	0.36	**<0.001**	0.21	0.10	0.32	**<0.001**	0.11	0.00	0.21	**0.042**	0.10	−0.01	0.21	***0.085***	0.09	−0.02	0.20	0.109

*Values presented are the standardized ß regression coefficients and 95% CIs. *Model 1* was unadjusted. *Model 2* was adjusted for the CAIDE–APOE-ε4 risk index. *Model* 3 was adjusted for age, race/ethnicity, sex, and level of education attained. *Model 4* included the variables of *Model 3* in addition to prevalent CV and AF events, CV risk factors (usage of anti-hypertensive and lipid lowering medications, systolic BP, diabetes mellitus status, total cholesterol, high-density lipoprotein cholesterol, pack-years of cigarette smoking), alcohol consumption, weekly amounts of moderate and vigorous physical activity, depression, usage of antidepressant medications, total gross family income ≥ $75,000 and mean HR. *Model 5* included the variables of *Model 4* and NT-proBNP. The value in parenthesis after each independent variable (PIP, PNNSS and PNNLS) is its SD. Statistically significant associations (*p* value < 0.05) are highlighted in bold. Borderline significant associations (0.05 ≤ *p* value ≤ 0.1) are highlighted using bold, italicized and underlined fonts.*

*AF, atrial fibrillation; ß, standardized linear regression coefficient; BP, blood pressure; CAIDE, Cardiovascular Risk Factors, Aging, and Incidence of Dementia; CASI, the Cognitive Abilities Screening Instrument score; CI, confidence interval; CV, cardiovascular; DS, digit span; DSC, digit symbol coding; HR, heart rate; HRF, heart rate fragmentation; PIP, percentage of inflection points; PNNLS, percentage of NN intervals in long (≥3) accelerative/decelerative segments; PNNSS, percentage of NN intervals in short (<3) accelerative/decelerative segments; NT-proBNP, N-terminal prohormone B-type natriuretic peptide; SD, standard deviation.*

The CAIDE–APOE-ε4 risk index attenuated the associations between HRF indices and the cognitive test scores only slightly. In contrast, age, sex, race and education reduced the strength of these associations between 40% and 70%. Additional adjustments had only a relatively small effect. Consistent with these observations is the finding that the adjusted R^2^ values of *Models 4* (0.325) and 5 (0.326) were only 2% higher than that of *Model 3* (0.307). Of note, despite including information on the strongest predictors of cognitive performance, namely age, sex and education, the model with the CAIDE–APOE-ε4 risk index had an adjusted R^2^ of only 0.072 (75% lower than that of *Model 3*).

#### Heart Rate Variability

In fully adjusted models, AVNN (inversely related to mean HR) was positively associated with CASI (*p* < 0.10) and the DSC (*p* < 0.05) scores (Appendix Table 1). None of the variability measures (rMSSD, SDNN and HF) was associated with any of the cognitive scores in the fully adjusted models. There were some significant or borderline significant associations in *Models 1–3*. However, for both rMSSD and HF power, these associations were negative, i.e., higher variability was associated with lower cognitive scores. Higher SDNN was associated with higher CASI scores solely in analyses adjusted for CAIDE–APOE-ε4.

### Prospective Association of Baseline HRF and HRV Indices With Exam 6 Cognitive Scores

#### Heart Rate Fragmentation

Heart rate fragmentation indices were associated (Table 4) with the CASI (*p* < 0.05), DSC (*p* < 0.05), DS forward (*p* < 0.1) and DS backward (*p* < 0.05) scores obtained at *exam 6*, 6.4 ± 0.5 (mean ± SD) years after *exam 5*. In fully adjusted analyses, a one-SD increase in PNNSS (13.7%) was associated with −0.61 (95% CI: −1.07 to −0.14), −1.54 (−2.54 to −0.54), −0.14 (−0.31 to 0.02) and −0.17 (−0.31 to −0.04) points decrease in the CASI, DSC, DS forward and DS backward scores, respectively.

**TABLE 4:** Multivariable linear regression analyses of the associations between the HRF indices (PIP, PNNSS and PNNLS) and the cognitive scores (CASI, DSC, DS forward and DS backward) measured at *exam 6*.

EXAM 6	MODEL 1				MODEL 2				MODEL 3				MODEL 4				MODEL 5			
	Unadjusted				CAIDE–APOE-ε4				Age, sex, race, education				Demographic + APOE-ε4 + CV				Model 4 + NT-proBNP			
													and other “traditional” risk factors							
	β	95% CI		*p*	β	95% CI		*p*	β	95% CI		*p*	β	95% CI		*p*	β	95% CI		*p*
**CASI**																				
PIP (7.01%)	−1.49	−2.00	−0.99	<0.001	−1.46	−1.96	−0.96	<0.001	−0.60	−1.05	−0.15	0.010	−0.50	−0.95	−0.04	0.032	−0.47	−0.92	−0.01	0.045
PNNSS (13.7%)	−1.53	−2.05	−1.01	<0.001	−1.43	−1.93	−0.92	<0.001	−0.68	−1.15	−0.21	0.005	−0.61	−1.07	−0.14	0.010	−0.57	−1.03	−0.10	0.018
PNNLS (12.6%)	1.54	1.02	2.06	<0.001	1.44	0.94	1.95	<0.001	0.69	0.22	1.16	0.004	0.57	0.11	1.02	0.015	0.52	0.06	0.98	0.026

**DSC**																				

PIP (6.95%)	−4.28	−5.37	−3.19	<0.001	−4.21	−5.29	−3.13	<0.001	−1.37	−2.29	−0.44	0.004	−1.17	−2.17	−0.18	0.021	−1.16	−2.15	−0.16	0.023
PNNSS (13.7%)	−3.91	−5.01	−2.80	<0.001	−3.67	−4.77	−2.57	<0.001	−1.60	−2.51	−0.69	0.001	−1.54	−2.54	−0.54	0.003	−1.55	−2.56	−0.54	0.003
PNNLS (12.6%)	3.97	2.86	5.08	<0.001	3.75	2.65	4.86	<0.001	1.59	0.68	2.51	0.001	1.46	0.47	2.45	0.004	1.47	0.48	2.47	0.004

**DS forward**																				

PIP (7.09%)	−0.29	−0.44	−0.13	<0.001	−0.26	−0.42	−0.11	0.001	−0.16	−0.31	0.00	0.049	−0.15	−0.31	0.02	*0.084*	−0.16	−0.32	0.01	*0.064*
PNNSS (13.7%)	−0.25	−0.41	−0.09	0.002	−0.22	−0.38	−0.06	0.008	−0.15	−0.30	0.00	*0.057*	−0.14	−0.31	0.02	*0.092*	−0.16	−0.33	0.01	*0.064*
PNNLS (12.6%)	0.26	0.10	0.42	0.002	0.23	0.07	0.39	0.006	0.15	0.00	0.31	*0.051*	0.14	−0.02	0.31	*0.092*	0.16	−0.01	0.32	*0.062*

**DS backward**																				

PIP (7.09%)	−0.30	−0.43	−0.17	<0.001	−0.28	−0.40	−0.15	<0.001	−0.14	−0.26	−0.01	0.037	−0.16	−0.30	−0.03	0.019	−0.16	−0.29	−0.02	0.024
PNNSS (13.7%)	−0.29	−0.43	−0.16	<0.001	−0.26	−0.39	−0.12	<0.001	−0.13	−0.25	−0.01	0.039	−0.17	−0.31	−0.04	0.009	−0.18	−0.31	−0.05	0.009
PNNLS (12.6%)	0.30	0.16	0.43	<0.001	0.26	0.13	0.40	<0.001	0.13	0.01	0.26	0.030	0.17	0.04	0.30	0.010	0.17	0.04	0.30	0.010

*Values presented are the standardized ß regression coefficients and 95% CIs. *Model 1* was unadjusted. *Model 2* was adjusted for the CAIDE–APOE-ε4 risk index. *Model 3* was adjusted for age, race/ethnicity, sex, and level of education attained. *Model 4* included the variables of *Model 3* in addition to prevalent CV and AF events, CV risk factors (usage of anti-hypertensive and lipid lowering medications, systolic BP, diabetes mellitus status, total cholesterol, high-density lipoprotein cholesterol, pack-years of cigarette smoking), alcohol consumption, weekly amounts of moderate and vigorous physical activity, depression, usage of antidepressant medications, total gross family income ≥ $75,000 and mean HR. *Model 5* included the variables of *Model 4* and NT-proBNP. The value in parenthesis after each independent variable (PIP, PNNSS and PNNLS) is its SD. Statistically significant associations (*p* value < 0.05) are highlighted in bold. Borderline significant associations (0.05≤*p* value ≤0.1) are highlighted using bold, italicized and underlined fonts.*

*AF, atrial fibrillation; BP, blood pressure; CAIDE, Cardiovascular Risk Factors, Aging, and Incidence of Dementia; CASI, the Cognitive Abilities Screening Instrument score; CI, confidence interval; CV, cardiovascular; DS, digit span; DSC, digit symbol coding; HR, heart rate; HRF, heart rate fragmentation; PIP, percentage of inflection points; PNNLS, percentage of NN intervals in long (≥3) accelerative/decelerative segments; PNNSS, percentage of NN intervals in short (<3) accelerative/decelerative segments; NT-proBNP, N-terminal prohormone B-type natriuretic peptide.*

#### Heart Rate Variability

There were relatively few (13 out of 80) significant or borderline significant associations between the HRV indices and the cognitive scores (Appendix Table 2). Notably, in the most adjusted models, there was only one significant association. Slower HR (higher AVNN) was associated with higher DS backward scores.

### Association of Baseline HRF and HRV Indices With Changes (Exam 6–Exam 5) in Cognitive Test Scores

#### Heart Rate Fragmentation

The HRF indices were strongly associated with the changes in cognitive scores (Table 5). Overall, higher HRF, i.e., higher PIP and PNNSS, was associated with larger decrements in cognitive scores. Similarly, lower HRF (higher degree of HR fluency indicated by higher PNNLS values) was associated with smaller decrements. In fully adjusted analyses, the associations were strongest with the Δ DSC scores and weakest with the Δ DS forward scores.

**TABLE 5:** Multivariable linear regression analyses of the associations between the HRF indices (PIP, PNNSS and PNNLS) and the changes (*exam 6 – exam 5*) in cognitive test scores (Δ CASI, Δ DSC, Δ DS forward and Δ DS backward).

Exam 6 – Exam 5	MODEL 1				MODEL 2				MODEL 3				MODEL 4				MODEL 5			
	Baseline cognitive scores				Baseline cognitive scores +				Baseline cognitive scores + age,				Model 3 + APOE-ε4 + CV				Model 4 + NT-proBNP			
					CAIDE–APOE-ε4				sex, race, education				and other “traditional” risk factors							
	β	95% CI		*p*	β	95% CI		*p*	β	95% CI		*P*	β	95% CI		*p*	β	95% CI		*p*
**Δ CASI**																				
PIP (7.01%)	−0.96	−1.35	−0.57	<0.001	−0.96	−1.36	−0.57	<0.001	−0.44	−0.83	−0.05	0.029	−0.37	−0.77	0.03	*0.071*	−0.34	−0.74	0.06	*0.096*
PNNSS (13.7%)	−0.97	−1.39	−0.54	<0.001	−0.94	−1.36	−−0.53	<0.001	−0.54	−0.96	−0.12	0.012	−0.44	−0.86	−0.03	0.037	−0.42	−0.84	0.00	*0.050*
PNNLS (12.6%)	0.99	0.57	1.40	<0.001	0.97	0.56	1.38	<0.001	0.55	0.13	0.96	0.010	0.42	0.01	0.83	0.045	0.39	−0.02	0.80	*0.063*

**Δ DSC**																				

PIP (7.02%)	−1.95	−2.71	−1.18	<0.001	−1.96	−2.72	−1.20	<0.001	−0.99	−1.73	−0.24	0.009	−1.06	−1.84	−0.27	0.008	−0.97	−1.76	−0.18	0.017
PNNSS (13.7%)	−1.64	−2.42	−0.86	<0.001	−1.59	−2.36	−0.82	<0.001	−0.95	−1.69	−0.22	0.011	−1.04	−1.81	−0.28	0.008	−1.00	−1.78	−0.22	0.012
PNNLS (12.6%)	1.65	0.87	2.43	<0.001	1.61	0.84	2.38	<0.001	0.95	0.22	1.68	0.011	1.01	0.26	1.77	0.009	0.96	0.18	1.73	0.016

**Δ DS forward**																				

PIP (7.02%)	−0.22	−0.34	−0.09	0.001	−0.21	−0.33	−0.09	0.001	−0.15	−0.28	−0.02	0.022	−0.13	−0.26	0.01	*0.067*	−0.13	−0.27	0.00	*0.058*
PNNSS (13.7%)	−0.20	−0.32	−0.07	0.002	−0.18	−0.31	−0.06	0.004	−0.14	−0.27	−0.02	0.025	−0.13	−0.27	0.01	*0.063*	−0.14	−0.28	0.01	*0.061*
PNNLS (12.6%)	0.20	0.08	0.32	0.001	0.19	0.07	0.31	0.003	0.15	0.02	0.27	0.022	0.13	0.00	0.27	*0.057*	0.14	0.00	0.28	*0.055*
**Δ DS backward**																				
PIP (7.09%)	−0.15	−0.26	−0.05	0.005	−0.15	−0.25	−0.04	0.007	−0.07	−0.18	0.05	0.243	−0.11	−0.22	0.01	*0.079*	−0.10	−0.22	0.02	0.102
PNNSS (13.7%)	−0.16	−0.27	−0.06	0.003	−0.15	−0.26	−0.04	0.006	−0.08	−0.19	0.02	0.130	−0.14	−0.25	−0.03	0.015	−0.14	−0.25	−0.03	0.016
PNNLS (12.6%)	0.17	0.06	0.28	0.002	0.16	0.05	0.26	0.004	0.09	−0.02	0.19	0.103	0.14	0.03	0.25	0.015	0.14	0.03	0.25	0.016

*Values presented are the standardized ß regression coefficients and 95% CIs. *Model 1* was unadjusted. *Model 2* was adjusted for the CAIDE–APOE-ε4 risk index. *Model 3* was adjusted for age, race/ethnicity, sex, and level of education attained. *Model 4* included the variables of *Model 3* in addition to prevalent CV and AF events, CV risk factors (usage of anti-hypertensive and lipid lowering medications, systolic BP, diabetes mellitus status, total cholesterol, high-density lipoprotein cholesterol, pack-years of cigarette smoking), alcohol consumption, weekly amounts of moderate and vigorous physical activity, depression, usage of antidepressant medications, total gross family income ≥ $75,000 and mean HR. *Model 5* included the variables in *Model 4* and NT-proBNP. The value in parenthesis after each independent variable (PIP, PNNSS and PNNLS) is its SD. Statistically significant associations (*p* value < 0.05) are highlighted in bold. Borderline significant associations (0.05≤*p* value ≤0.1) are highlighted using bold, italicized and underlined fonts.*

*AF, atrial fibrillation; BP, blood pressure; CAIDE, Cardiovascular Risk Factors, Aging, and Incidence of Dementia; CASI, the Cognitive Abilities Screening Instrument score; CI, confidence interval; CV, cardiovascular; DSC, digit symbol coding; DS, digit span; HR, heart rate; PIP, percentage of inflection points; PNNLS, percentage of NN intervals in long (≥3) accelerative/decelerative segments; PNNSS, percentage of NN intervals in short (<3) accelerative/decelerative segments; NT-proBNP, N-terminal prohormone B-type natriuretic peptide.*

**FIGURE 2:**
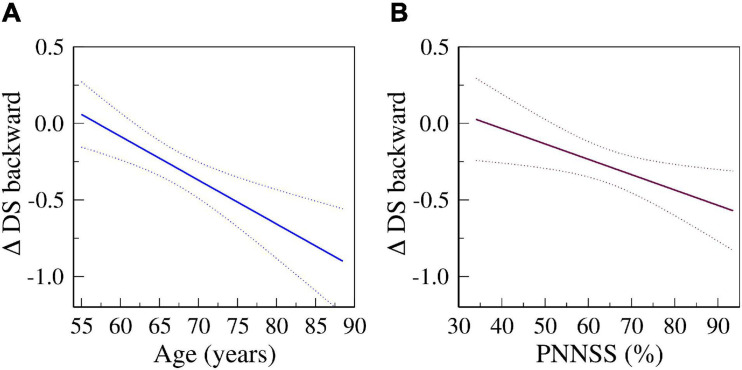
Predicted changes in DS backward scores from *exams 5* to *6* as a function of age (A) and of PNNSS values (B) derived from fully adjusted models (*Model 4*). Solid and dotted lines are the predicted and 95% confidence interval values, respectively.

In fully adjusted models, a one-SD increase in PNNSS was associated with a decrease of −0.44 (95% CI: −0.86 to −0.03), −1.04 (−1.81 to −0.28), −0.13 (−0.27 to 0.01) and −0.14 (−0.25 to −0.03) points in the CASI, DSC, DS forward and DS backward scores, respectively. Figure 2 depicts the predicted changes in DS backward scores derived from fully adjusted analyses as a function of the participants’ age (left) and baseline PNNSS (right). The representative graphs show that cognitive decline *accelerated* with the participants’ age as well as with baseline degree of HRF. In the time period between *exams 5* and *6*, cognitive performance decreased more for older than younger participants. Similarly, in the same time period, cognitive performance decreased more for participants with higher degrees of HRF. Of note, the associations between HRF and the changes in cognitive scores were not dependent on the participants’ age (the *p*-value for the interaction term of HRF and age was not significant in any model).

#### Heart Rate Variability

There were few (5 out of 80) significant or borderline associations between HRV indices and the longitudinal changes in cognitive scores (Appendix Table 3). In fully adjusted analyses, there was only one significant result. Namely, slower HR was associated with smaller decreases in the DS backward scores.

**FIGURE 3:**
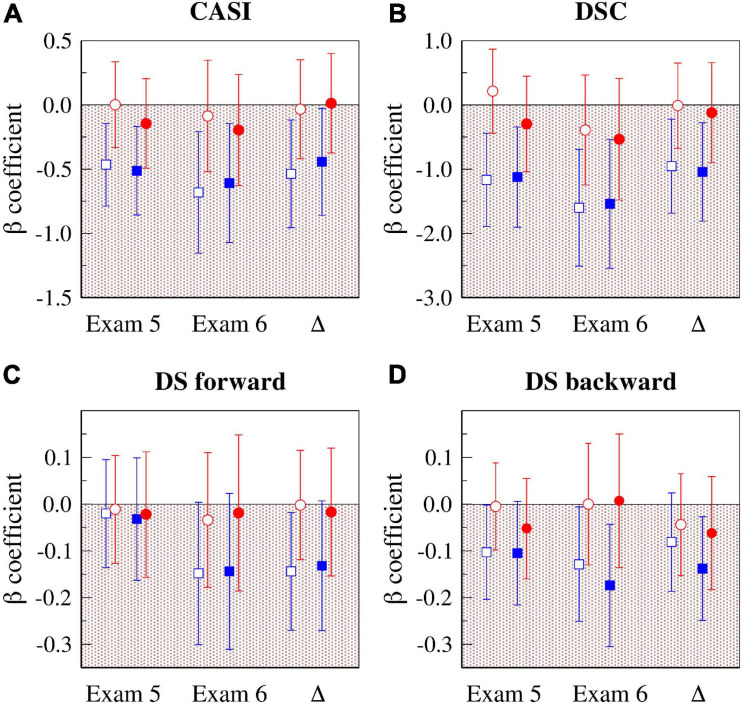
Associations of HR dynamical indices (HRF: PNNSS and HRV: rMSSD) with cognitive test scores at exams 5 and 6, and with their longitudinal changes (Δ = *exam 6* – *exam 5*). The graphs show the standardized ß coefficients for the associations of CASI (A), DSC (B), DS forward (C) and DS backward (D) with PNNSS (blue symbols) and rMSSD (red symbols). The open symbols are the standardized ß coefficients from models adjusted for age, sex, race and education. The solid symbols are the standardized ß coefficients from fully adjusted models (*Model 4*). The error bars are the 95% CIs. CASI, the Cognitive Abilities Screening Instrument score; DSC, digit symbol coding; DS, digit span; CI, confidence interval; HR, heart rate; HRF, heart rate fragmentation; HRV, heart rate variability; PNNSS, percentage of NN intervals in short (<3) accelerative/decelerative segments; rMSSD, root mean square of successive NN interval differences.

Figure 3 presents a graphical summary of the results of two sets of analyses described above, one adjusted for age, sex, race and education (*Model 3*, open symbols), and the other “fully” adjusted (*Model 4*, solid symbols). Specifically, the figure shows the standardized ß coefficients for the associations of PNNSS (blue symbols) and rMSSD (red symbols) with cognitive scores at exams 5 and 6 and the changes in cognitive scores.

Table 6 shows the regression coefficients for all the variables included in the fully adjusted models. Higher PNNSS was consistently associated (*p* < 0.05 or *p* < 0.1) with the lower cognitive scores at *exams 5* and *6* and with larger decrements in scores from *exams 5* to *6*. (The only exception was the cross-sectional association with DS forward.)

**TABLE 6:** Multivariable linear regression models of CASI, DSC, DS forward and backward scores at *exams 5* and *6* and of their changes from *exams 5* to *6.*

	EXAM 5								EXAM 6								EXAM 6 - EXAM 5							
Fully Adjusted Models	CASI		DSC		DS forward		DS backward		CASI		DSC		DS forward		DS backward		Δ CASI		Δ DSC		Δ DS forward		Δ DS backward	
*Variables*	β	*p*	β	*p*	β	*p*	β	*p*	β	*p*	β	*p*	β	p	β	*p*	β	*p*	β	*p*	β	*p*	β	*p*
PNNSS	−0.513	**0.004**	−1.123	**0.005**	−0.032	0.630	−0.105	***0.065***	−0.608	**0.010**	−1.540	**0.003**	−0.144	***0.092***	−0.174	**0.009**	0.443	**0.037**	1.043	**0.008**	0.132	***0.063***	0.138	**0.015**
Age	−1.324	**<0.001**	−5.525	**<0.001**	−0.257	**<0.001**	−0.187	**0.001**	−1.833	**<0.001**	−6.853	**<0.001**	−0.343	**<0.001**	−0.294	**<0.001**	1.560	**<0.001**	3.865	**<0.001**	0.224	**0.003**	0.243	**<0.001**
Male	−0.512	0.194	−5.522	**<0.001**	0.240	0.101	−0.135	0.265	−0.028	0.956	−5.718	**<0.001**	0.139	0.462	−0.298	***0.064***	−0.385	0.404	2.220	**0.021**	0.046	0.767	0.218	0.116
Race/ethnicity																								
White (reference)																								
Chinese−American	−2.839	**<0.001**	5.086	**<0.001**	2.192	**<0.001**	−0.026	0.899	−2.753	**<0.001**	1.222	0.453	1.707	**<0.001**	−0.192	0.436	1.245	***0.064***	2.463	***0.050***	−0.924	**0.002**	0.042	0.846
Black	−3.736	**<0.001**	−7.749	**<0.001**	−0.348	**0.027**	−1.119	**<0.001**	−2.971	**<0.001**	−8.479	**<0.001**	−0.591	**0.002**	−0.977	**<0.001**	1.104	**0.029**	3.443	**0.001**	0.455	**0.005**	0.433	**0.006**
Hispanic	−5.598	**<0.001**	−8.022	**<0.001**	−1.739	**<0.001**	−1.626	**<0.001**	−5.700	**<0.001**	−11.658	**<0.001**	−2.040	**<0.001**	−1.789	**<0.001**	2.996	**<0.001**	6.506	**<0.001**	1.154	**<0.001**	0.936	**<0.001**
Education																								
≤ High school (reference)																								
Some college	3.375	**<0.001**	8.727	**<0.001**	0.666	**<0.001**	0.898	**<0.001**	2.065	**0.004**	7.555	**<0.001**	0.434	***0.060***	0.590	**0.002**	−0.885	0.141	−3.128	**0.006**	−0.224	0.220	−0.237	0.133
≥ Bachelor degree	4.195	**<0.001**	11.45	**<0.001**	0.490	**0.004**	0.933	**<0.001**	2.646	**<0.001**	9.280	**<0.001**	0.339	0.141	0.783	**<0.001**	−0.864	0.133	−2.495	**0.029**	−0.213	0.243	−0.381	**0.013**
APOE-ε4	−0.703	***0.057***	−0.820	0.311	−0.231	0.100	−0.111	0.346	−0.897	***0.056***	−2.980	**0.005**	−0.324	***0.059***	−0.071	0.622	0.897	**0.033**	1.454	0.100	0.137	0.312	0.056	0.651
AVNN	0.450	**0.011**	1.176	**0.002**	0.038	0.573	0.106	***0.073***	0.430	***0.051***	0.801	0.115	0.159	***0.081***	0.227	**0.002**	−0.214	0.292	−0.542	0.172	−0.107	0.151	−0.178	**0.005**
Systolic BP	−0.259	0.158	−0.597	0.139	0.014	0.830	−0.050	0.347	−0.612	**0.028**	−0.923	***0.087***	0.015	0.862	−0.103	0.142	0.433	***0.079***	0.888	**0.040**	−0.052	0.459	0.079	0.172
Anti-hypertensive Rx	−0.183	0.621	−2.170	**0.008**	−0.044	0.747	−0.212	***0.071***	−0.462	0.293	−0.767	0.456	−0.068	0.698	−0.118	0.439	0.128	0.744	−0.602	0.455	0.054	0.709	0.058	0.657
Diabetes	−0.226	0.640	−2.350	**0.026**	−0.454	**0.011**	−0.111	0.463	−1.992	**0.006**	−2.894	**0.045**	−0.418	***0.062***	−0.094	0.618	1.449	**0.018**	0.631	0.582	0.159	0.368	0.077	0.628
Total cholesterol	−0.030	0.870	−0.430	0.300	0.043	0.547	0.082	0.182	−0.150	0.494	−0.067	0.896	0.127	0.155	0.038	0.612	−0.051	0.791	−0.372	0.365	−0.074	0.341	0.005	0.940
HDL	−0.104	0.562	0.322	0.418	0.057	0.424	0.006	0.932	−0.318	0.190	0.021	0.966	−0.041	0.648	−0.013	0.874	0.104	0.627	−0.040	0.920	0.127	0.101	−0.001	0.984
Lipid lowering Rx	−0.300	0.443	0.693	0.410	0.038	0.790	−0.102	0.410	−0.515	0.273	0.277	0.806	−0.016	0.928	−0.110	0.478	0.225	0.579	−0.002	0.998	0.025	0.869	−0.080	0.555
Pack−years of cigarette smoking	0.705	**<0.001**	0.336	0.372	0.015	0.806	0.040	0.465	0.339	0.103	0.178	0.716	0.093	0.252	0.063	0.391	0.084	0.649	−0.062	0.873	−0.083	0.213	−0.077	0.222
Prevalent CVEs & AF	−0.233	0.666	−2.677	**0.031**	−0.023	0.900	0.059	0.723	0.116	0.882	−1.314	0.457	0.340	0.139	0.055	0.835	−0.032	0.961	−1.604	0.234	−0.251	0.233	−0.121	0.581
Physical activity, ln	−0.215	0.243	0.278	0.471	−0.044	0.506	−0.180	**0.001**	−0.401	***0.068***	−0.857	***0.095***	−0.197	**0.013**	−0.237	**0.001**	0.211	0.283	0.801	***0.057***	0.098	0.163	0.143	**0.018**
Depression, CESD ≥ 16	−1.974	**<0.001**	−2.803	**0.009**	−0.306	***0.098***	−0.232	0.144	−1.172	***0.074***	−2.147	0.160	−0.114	0.594	−0.076	0.702	0.254	0.640	0.665	0.560	0.030	0.866	0.101	0.549
Anti-depression Rx	0.724	0.110	0.534	0.630	0.305	***0.089***	0.331	**0.037**	0.449	0.427	−1.402	0.344	0.168	0.480	0.228	0.313	0.041	0.937	2.092	***0.079***	0.039	0.852	−0.008	0.968
Alcohol use	1.632	**<0.001**	3.635	**<0.001**	0.431	**0.002**	0.397	**0.001**	2.494	**<0.001**	1.684	***0.099***	0.308	***0.073***	0.475	**0.001**	−1.984	**<0.001**	0.405	0.627	−0.069	0.633	−0.221	***0.074***
Gross income ≥ 75K	0.776	**0.028**	3.646	**<0.001**	0.220	0.156	0.463	**0.001**	0.367	0.381	4.025	**<0.001**	0.167	0.370	0.343	**0.033**	0.111	0.772	−2.286	**0.011**	−0.010	0.948	−0.070	0.615
Baseline cognitive scores (6.9/17.1/2.7/2.5)																	3.548	**<0.001**	5.694	**<0.001**	1.279	**<0.001**	1.298	**<0.001**

*Values shows are the standardized ß regression coefficients and *p* values for each one of the variables in the fully adjusted models. The SDs for each of the continuous independent variables in the models are: PNNSS — 13.7%, age — 8.95 years, AVNN — 134 ms, systolic BP — 20.1 mmHg, total cholesterol — 36.8 mg/dl, HDL — 16.4 mg/dl); pack-years of cigarette smoking – 1.49, physical activity (ln) — 1.06 MET-min/wk, CASI — 6.0, DSC — 17.1, DS forward — 2.7, and DS backward — 2.5. Statistically significant associations (*p* value < 0.05) are highlighted in bold. Borderline significant associations (0.05 ≤ *p* value ≤ 0.1) are highlighted using bold, italicized and underlined fonts.*

*AF, atrial fibrillation; AVNN, average value of the NN intervals; BP, blood pressure; CASI, the Cognitive Abilities Screening Instrument score; CESD, Center for Epidemiological Studies Depression scale; CVE, cardiovascular event; DSC, digit symbol coding; DS, digit span; HDL, high-density lipoprotein cholesterol; HR, heart rate; MET, metabolic equivalent; PNNSS, percentage of NN intervals in short (<3) accelerative/decelerative segments; Rx, medication.*

Of note, further adjusting the cross-sectional and longitudinal analyses described above (*Model 4*) for CAC, measures of sleep structure (the percentage of time in REM and the percentage time in SWS), a measure of sleep disorder breathing (AHI), and measures of hypoxemia (ODI and the % of time SpO_2_ < 90%) did not qualitatively change our results (not shown). Finally, we note that no interactions by sex or race were detected in any of the cross-sectional or the longitudinal analyses previously described.

## Discussion

The present investigation was designed to evaluate cross-sectional and prospective associations between HRF, a newly identified property of cardiac neuroautonomic dynamics, and cognitive function in the large MESA cohort of those who underwent a PSG study. The degree of HRF was assessed at *exam 5* (our baseline) using previously described metrics (Costa et al., 2017a,b). Cognitive performance was assessed at this baseline and 6.4 ± 0.5 years later at *exam 6*, using the CASI, DSC and DS forward and backward tests, which are designed to assess overall cognitive performance, processing speed, short-term memory and working memory, respectively.

Our key findings were: (1) in cross-sectional analyses, HRF was inversely associated with all cognitive scores other than the DS forward scores (higher HRF, lower scores); (2) in prospective analyses, HRF was inversely associated with all cognitive scores; (3) HRF was associated with the *changes* in all cognitive scores (higher baseline HRF, larger decrement in scores); (4) HRF metrics added predictive value to the CAIDE–APOE-ε4 risk index and to models with a combination of risk factors for cognitive impairment including NT-proBNP serum concentration; (5) slower HR was cross-sectionally associated with the CASI and DSC scores, and prospectively associated with the DS backward scores; and (5) the HRV indices rMSSD, SDNN and HF power were not cross-sectionally or longitudinally associated with cognitive scores.

Overall, our results indicate that an *increased degree of HRF was associated with both worse concurrent and future cognitive function*. Our analyses also showed that *increased baseline HRF was associated with steeper future cognitive decline*. For example, in fully adjusted analyses (Figure 2), participants whose HRF at baseline (PNNSS) was 80% *vs.* 60% lost twice the number of points in the DS backward test from *exams 5* to *6* [0.44 (0.27–0.60) *vs.* 0.23 (0.12–0.35) points]. Similarly, we found that older age was associated with greater cognitive decline – the older the participants the larger the longitudinal decrease in cognitive scores. For example, in fully adjusted analyses (Figure 2), the decrease in DS backward scores from *exams 5* to *6* for participants aged 75 *vs.* 65 was approximately twice as high [0.51 (0.35–0.68) *vs.* 0.23 (0.11–0.34) points]. The results are consistent with the *acceleration* of cognitive decline with aging as well as with baseline degree of HRF.

In both cross-sectional and longitudinal analyses, HRF added predictive value to the CAIDE–APOE-ε4 dementia risk index (Kivipelto et al., 2006; Exalto et al., 2014; Rundek et al., 2020). This index was developed to identify individuals at increased risk for dementia. It was validated in a multiethnic population in the United States (Exalto et al., 2014). The risk index is based on age, sex, BMI, systolic BP, total cholesterol, physical activity, educational level and APOE-ε4 status. Notably, the strength of the associations between cognitive scores and HRF indices were minimally attenuated by the inclusion in the models of this widely used risk index for dementia. This finding highlights the independence (“orthogonality”) of the two quantities, HRF from CAIDE-APOE-ε4.

We found that HRF was significantly associated with cognitive performance (higher HRF, worse cognitive performance) independent of a combination of widely acknowledged risk factors of cognitive impairment including demographic (age, sex), socioeconomic (level of education, gross family income), genomic (APOE-ε4 allele), behavioral (smoking, alcohol consumption, degree of physical activity), mental health (depression) variables, and CV risk factors (hypertension, hyperlipidemia, diabetes and CAC). Moreover, HRF added predictive value to models that in addition to the variables mentioned above also included serum concentration of NT-proBNP. This analyte has been reported to be associated with incident dementia in MESA (Ostovaneh et al., 2020) and other studies (Nagata et al., 2019), with MRI measures of cognitive function and structure (Zonneveld et al., 2017; Ferguson et al., 2018), as well as, with overall or some domains of cognitive impairment (Kerola et al., 2010; Ferguson et al., 2018; Gallo et al., 2020).

Our findings are consistent with proposed links between cardiac neuroautonomic regulation and cognitive function (Collins et al., 2012). Although epidemiologic studies such as the present one cannot resolve the complex and likely multifactorial underpinnings of this relationship, the findings are supportive of a number of mechanistic considerations. First, there is evidence that central nervous system dysfunction is closely linked to both clinical atherosclerotic disease and multiple CV risk factors (Newman et al., 2005; Gorelick et al., 2011). Our previous studies (Costa et al., 2018, 2021) in MESA also supported this link. We found that high HRF was predictive of major adverse CVEs and AF, which are themselves associated with advanced cognitive impairment and dementia (Deckers et al., 2017). We adjusted the analyses for common CV risk factors in addition to other potential confounders such as CAC (a marker of sustained, end-stage inflammation and severe vascular disease). Of note, to the extent that CV disease processes are only partially captured by the variables above, the possibility remains that preclinical or clinical CV disease markers still partially mediate the associations between HRF and cognitive performance.

A second and related possibility is that cardiac neuroautonomic impairment affects cognitive performance via its contribution to arterial BP dysregulation, encompassing but not limited to degraded baroreflex sensitivity (Meel-van den Abeelen et al., 2013). The resulting hemodynamic instability may manifest as increased BP fluctuations leading to suboptimal cerebral perfusion (Collins et al., 2012; Wolters et al., 2016; Schaich et al., 2020). Dysregulation of BP has been linked to cerebrovascular disease and related brain pathology (e.g., white matter lesions and lacunar infarctions) (Gómez-Angelats et al., 2004), factors that may further degrade cardiac neuroautonomic control. The result of these interconnected processes may be a vicious cycle whereby impaired cerebral autoregulation begets further cognitive impairments.

A third putative link between cardiac autonomic dysfunction and cognitive impairment is via pathologic alterations in neuro-immunomodulation. The role of the vagus in regulating systemic inflammatory response and tissue damage at multiple anatomic sites has emerged as a major area of investigation (Sartori et al., 2012; Pavlov and Tracey, 2017). The salutary and likely complex effects of vagal activation in suppressing excessive cytokine activation and other mediators of inflammation have been ascribed to a “cholinergic anti-inflammatory” network (Pavlov and Tracey, 2005, 2017; Bonaz et al., 2016). Conversely, abnormally decreased vagal activity, of which increased HRF is a marker, may mediate proinflammatory diatheses affecting the central nervous system. In the most severe cases, these inflammatory processes may contribute to the pathogenesis of Alzheimer’s disease and related dementias (Jessen et al., 2010).

Of further note, evidence that parasympathetic disruption may be initiated and perpetuated by peripheral and central neurodegenerative changes seen with aging, CV disease, as well as subclinical Alzheimer’s disease, suggests that HRF may have additional value as a predictor of incident dementias.

For comparison of performance and translational value, we included traditional HRV metrics in our study. Traditional indices of HR variability and measures of HRF assess different mathematical properties of HR dynamics. The former measures the amplitude of beat-to-beat fluctuations in HR. The latter quantifies the frequency of reversals in acceleration, i.e., of the changes from HR acceleration (when the difference between consecutive NN intervals contracts) to deceleration (expands) and vice-versa. In this study, we found that the *amplitude* of HR fluctuations, as quantified by traditional HRV metrics, was not consistently associated with cognitive performance. Other large epidemiologic studies have also investigated the associations between HRV indices, as putative measures of neuroautonomic function, and cognitive test scores. The results are difficult to reconcile. Some report positive associations (Britton et al., 2008; Frewen et al., 2013; Mahinrad et al., 2016; Schaich et al., 2020) between selected HRV metrics and specific cognitive test scores; others, like ours, find no consistent associations (Kim et al., 2006; Collins et al., 2012).

These discrepancies are likely due to the limitations of traditional HRV metrics themselves (Costa et al., 2017a; Hayano and Yuda, 2019; Hayano et al., 2020), namely the fact that fragmentation of HR dynamics instead of physiologic vagal tone modulation may inflate the value of HRV metrics. This concern is especially pertinent in populations of middle age to older individuals, such as the MESA cohort. Additionally, differences in study populations, cognitive tests and methods of assessing HRV, which can be based on different metrics and may be derived from short (typically 10 s) or long (typically multiple hours) ECG recordings, may also contribute to the differences in findings.

As discussed in detail in references (Costa et al., 2017a, 2018, 2021; Costa and Goldberger, 2019) the two major sources of short-term cardiac interbeat interval variability, namely parasympathetic activity (vagal tone modulation) and HRF, are *not* distinguishable using traditional HRV measures. This limitation undermines the reliability and translational value of these metrics (Hayano and Yuda, 2019). The graphs of the cross-sectional relationships of rMSSD and SDNN with participants’ age (Figure 1) illustrate this major limitation. Vagal tone modulation is known to decrease with aging. If the amplitude of HR fluctuation quantified by rMSSD and SDNN were reliable measures of vagal tone modulation, they should also decrease with cross-sectional age. Instead, we found non-monotonic (U-shaped) relationships of rMSSD and SDNN with age (Figure 1). Similar results were obtained for HF power (not shown). The U-shaped relationships between HRV metrics and age have been noted by others (Almeida-Santos et al., 2016; Hayano et al., 2020). In contrast, the HFR metrics, PIP and PNNSS, increased monotonically (and linearly) with cross-sectional age (Figure 1). (The PNNLS index [whose values decrease as fragmentation increases] monotonically decreased with cross-sectional age [not shown].) Consistent with a previous MESA study (Schaich et al., 2020), we found that slower HR, a global marker of relative parasympathetic predominance, was associated with higher cognitive performance.

In an era of ECG wearables technology, a dynamical risk marker such as HRF may be especially attractive given the fact that it is computationally inexpensive and it can be continuously updated. In this context, HRF may also have important applications in monitoring and prediction of the safety and efficacy of therapeutic interventions: those that increase HRF will potentially be of concern. The results presented here in conjunction with those reported elsewhere (showing that increased HRF was a precursor of incident major adverse CV (Costa et al., 2018) and AF (Costa et al., 2021) events), suggest that HRF may help define populations that would benefit from early initiation of therapeutic interventions aimed at delaying/reversing cognitive decline and/or preventing adverse CVEs, themselves associated with worse cognition.

## Limitations of the Study

In MESA, assessment of cognitive function is limited to three standard tests, CASI, DSC and DS, which only probe selected cognitive domains. Another limitation is the fact that despite adjusting our analyses for demographic, socioeconomic and behavioral variables, in addition to socioeconomic status, traditional CV risk factors and APOE*-*ε4 allele carriage, the possibility of residual confounding could not be excluded. However, we did try to minimize such possibility by further adjusting the models for CAC, NT-proBNP, sleep structure and hypoxemia variables, which have themselves been associated with incident CV and AF events and/or cognitive decline. We note that the participants who underwent cognitive testing at *exam 6* (only 53% of those in our MESA-Sleep study cohort) were on average three years younger and overall healthier than those who did not. Furthermore, at *exam 5*, participants who would be re-tested at *exam 6* performed significantly better in all cognitive tests. Despite this selection bias and the lower statistical power for the longitudinal analyses compared to the cross-sectional ones, our results were still significant. Our findings will need to be validated in other populations.

## Conclusion

In MESA, higher degree of HRF, a marker of cardiac neuroautonomic impairment, was cross-sectionally and prospectively associated with worse cognitive performance. Furthermore, cognitive decline; accelerated with age as well as baseline degree of HRF (the higher HRF at baseline, the greater the loss of cognitive performance during follow-up). The potential translational utility of these findings is enhanced by the observation that HRF added predictive value to well-known risk factors of cognitive decline, such as age and subclinical CV risk markers.

## Data Availability Statement

The data analyzed in this study is subject to the following licenses/restrictions: MESA data can be accessed after submitting a manuscript proposal to MESA Publications & Presentations Committee and obtaining its approval to conduct the research work. Requests to access these datasets should be directed to Karen Hansen, hansenk3@u.washington.edu.

## Ethics Statement

Institutional review boards approved the conduct of the study. Written informed consent was obtained from all participants.

## Author Contributions

MC, SH, and AG: conception and research design. SR (PSG), SH, and TH (cognitive scores): data acquisition. MC: data analysis and preparation of tables and figures. MC and AG: manuscript drafting. All authors: interpretation of results and manuscript editing.

## Conflict of Interest

SR reports consulting fees from Apnimed Inc., Jazz Pharma and Eisai Inc., unrelated to this work, grant funding from Jazz Pharma unrelated to this work and receipt of loaned equipment from Nox Medical Inc. and Philips Respironics for use in an NIH trial. The remaining authors declare that the research was conducted in the absence of any commercial or financial relationships that could be construed as a potential conflict of interest.

## Publisher’s Note

All claims expressed in this article are solely those of the authors and do not necessarily represent those of their affiliated organizations, or those of the publisher, the editors and the reviewers. Any product that may be evaluated in this article, or claim that may be made by its manufacturer, is not guaranteed or endorsed by the publisher.

## Footnotes

1 CHARGE-AF, Cohorts for Heart and Aging Research in Genomic Epidemiology-Atrial Fibrillation.

## Supplementary Material

Supplementary Table 1 Multivariable linear regression analyses of the associations between the HRV indices (AVNN, rMSSD, SDNN and HF power) and the cognitive scores (CASI, DSC and DS) measured at *exam 5*.

Supplementary Table 2 Multivariable linear regression analyses of the associations between the HRV indices (AVNN, rMSSD, SDNN and HF power) and the cognitive scores (CASI, DSC and DS) measured at *exam 6*.

Supplementary Table 3 Multivariable linear regression analyses of the associations between the HRV indices (AVNN, rMSSD, SDNN and HF) and the changes (*exam 6* – *exam 5*) in cognitive scores (Δ CASI, Δ DSC, Δ DS forward and Δ DS backward).

## Abbreviations

AF: atrial fibrillation
AVNN: average of all NN intervals
BMI: body mass index
BP: blood pressure
CAC: coronary artery calcification
CAIDE: Cardiovascular Risk Factors, Aging, and Incidence of Dementia
CASI: Cognitive Abilities Screening Instrument
CHARGE: Cohorts for Heart and Aging Research in Genomic Epidemiology
CV: cardiovascular
CVE: cardiovascular event
DS: digit span
DSC: digit symbol coding
ECG: electrocardiogram/electrocardiographic
HF, high-frequency power: the total spectral power of all NN intervals between 0.15 and 0.40 Hz
HR: heart rate
HRF, heart rate fragmentation metric: the percentage of Δ NN that belong to long (≥ 3 Δ NN) accelerative and decelerative segments
HRV: heart rate variability
MESA: Multi-Ethnic Study of Atherosclerosis
N: normal sinus beat
NN: normal-to-normal sinus interbeat interval
NT-proBNP: N-terminal prohormone B-type natriuretic peptide
ODI: oxygen desaturation index
PIP: percentage of inflection points
PNNLS: percentage of Δ NN (≥ 3) intervals in long accelerative/decelerative segments
PNNSS: percentage of Δ NN intervals in short (< 3) accelerative/decelerative segments
PSG: polysomnographic study
REM: rapid eye movement
rMSSD: the root mean square of successive NN interval differences
SD: standard deviation
SDNN: mean of the SDs of NN intervals in all 5-min segments
SpO_2_: pulse oximeter oxygen saturation
SWS: slow wave sleep
WAIS-III: Wechsler Adult Intelligence Scale III.
